# Cephalad intertransverse process block for thoracic herpes zoster: a practical approach to enhance safety and effect

**DOI:** 10.1186/s40981-025-00801-z

**Published:** 2025-06-16

**Authors:** Keisuke Yoshida, Yoshie Noji, Ryosuke Sasaki, Atsuyuki Hosono, Satoki Inoue

**Affiliations:** Department of Anesthesiology, Fukushima Medical University School of Medicine, 1 Hikarigaoka, Fukushima City, Fukushima 960-1295 Japan

*To the Editor:*

Thoracic herpes zoster often presents as unilateral localized lesions along dermatomes. Therefore, neuraxial-adjacent nerve blocks, such as erector spinae plane block (ESPB), paravertebral block (PVB), and intertransverse process block (ITPB), are reasonable options for managing both acute and chronic pain. Recent meta-analyses have suggested that ESPB is safer than PVB regarding the treatment of acute pain associated with thoracic herpes zoster [1].

While PVB provides effective analgesia extending from the dorsal to the ventral regions, its deeper anatomical location raises safety concerns, including pneumothorax and bleeding complications in patients with bleeding tendencies. In contrast, ESPB is a more superficial technique with a lower risk of pneumothorax, although its efficacy in achieving anterior spread can be inconsistent [2]. ITPB offers a favorable balance, potentially providing more reliable anterior analgesia than ESPB with fewer complications compared to PVB [3].

We therefore propose performing ITPB at one or two vertebral levels cephalad to the affected dermatomes (Fig. 1) for the following reasons. First, ultrasound-guided blocks at the exact level of herpes zoster lesions should be avoided when possible, in order to minimize pain due to probe pressure and reduce the risk of infection if a rash is present. Second, the distribution of injected agents in paraspinal nerve blocks can be influenced by gravity [4]. This suggests that local anesthetic reaching the paravertebral space may spread in a caudal direction. Third, a three-dimensional micro-computed tomography study previously demonstrated that the retro-superior costotransverse ligament spaces—the ITPB target sites—are connected to the caudal paravertebral space via narrow anatomical slits [5]. Although the directional spread of local anesthetic into the paravertebral space following ITPB has not yet been clearly established in clinical studies, these anatomical findings may provide a theoretical basis for consideration of a slightly cranial approach relative to the intended dermatome level. Regarding this, Nielsen et al. reported that ITPB with 21 mL of 0.75% ropivacaine using a paramedian sagittal approach at the Th4 level with the patient in the sitting position resulted in a sensory block primarily between the T5 and T7 levels [6].

**Fig. 1:**
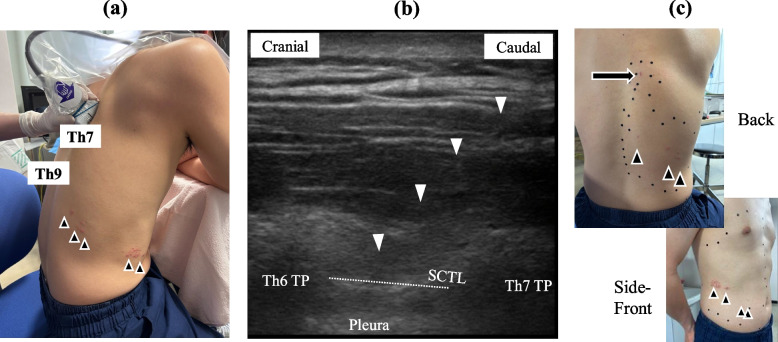
**a** One of the authors (KY), a man in his 30s, presented with acute herpes zoster involving the right posterior thoracic region around the Th9–Th10, extending to the lateral and anterior abdominal areas. His Numerical Rating Scale (NRS) score before ITPB was 3–4 out of 10. An ultrasound-guided ITPB was performed at the Th6/7 level using an in-plane approach in a sitting position. **b** Ultrasound image of ITPB performed using a paramedian sagittal approach. A linear probe was placed parallel to the spine at the Th6/7 level, slightly lateral to the transverse process. The needle (white arrowheads) was advanced using an in-plane approach. After skin infiltration with 2 mL of 1% lidocaine, 8 mL of 0.3% levobupivacaine was injected slightly dorsal to the superior costotransverse ligament (dotted line). **c** After the ITPB, the patient remained seated for 30 minutes. Subsequently, with the patient blindfolded to avoid visual bias, the area of hypoalgesia was assessed using a pinprick test, and was marked with small black stickers. The post-procedure NRS score was 0 out of 10. The area enclosed by the black stickers indicates the region of reduced pinprick sensation. The black arrow indicates the needle insertion site for the ITPB. The black arrowheads mark the location of the herpes zoster rash. NRS, Numerical Rating Scale; ITPB, intertransverse process block; SCTL, superior costotransverse ligament; TP, transverse process

Nerve blocks administered during the acute phase of herpes zoster have been shown to be effective not only for pain relief but also for reducing the incidence of postherpetic neuralgia [1]. We believe that our ITPB technique offers a practical approach that will contribute to both safer and more effective pain management in thoracic herpes zoster patients.

## Data Availability

Not applicable.
